# Epidemiology of COVID-19 Among Incarcerated Individuals and Staff in Massachusetts Jails and Prisons

**DOI:** 10.1001/jamanetworkopen.2020.18851

**Published:** 2020-08-21

**Authors:** Monik C. Jiménez, Tori L. Cowger, Lisa E. Simon, Maya Behn, Nicole Cassarino, Mary T. Bassett

**Affiliations:** 1 Division of Women’s Health, Brigham and Women’s Hospital, Boston, Massachusetts; 2 Department of Medicine, Harvard Medical School, Boston, Massachusetts; 3 Department of Epidemiology, Harvard T.H. Chan School of Public Health, Boston, Massachusetts; 4 François-Xavier Bagnoud Center for Health and Human Rights, Harvard T.H. Chan School of Public Health, Boston, Massachusetts; 5 Department of Oral Health Policy and Epidemiology, Harvard School of Dental Medicine, Boston, Massachusetts

## Abstract

This cohort study describes the COVID-19 burden among incarcerated individuals and staff in Massachusetts jails and prisons and assesses the association of COVID-19 case rates with decarceration and testing rates.

## Introduction

Incarcerated populations have exceptionally high risk of coronavirus disease 2019 (COVID-19) transmission and mortality due to overcrowding, movement through facilities, and high rates of chronic illness; hence, physical distancing is not a viable mitigation strategy.^1^ As of June 6, 2020, at least 42 107 cases and 510 deaths have occurred among individuals incarcerated in US prisons.^2^ Decarceration and increased testing may reduce transmission, but their efficacy is uncertain.^3^ Jails confine nearly one-third of incarcerated individuals, but data on COVID-19 in jails are limited. However, Massachusetts reports data on COVID-19 in both county jails and state prisons. We describe the COVID-19 burden in these settings and its association with decarceration and testing rates.

## Methods

Data used in this cohort study were reported by 16 Massachusetts Department of Corrections (MA DOC) facilities and 13 county-level systems from April 5 through July 8, 2020, pursuant to a court order.^4^ This study used publicly available, deidentified data and was deemed exempt from institutional review board approval by Partners HealthCare. We followed the Strengthening the Reporting of Observational Studies in Epidemiology (STROBE) reporting guideline.

We used baseline facility populations to calculate cumulative testing and laboratory-confirmed case rates per 1000 persons and changes in incarcerated population size (ie, decarceration). Case and testing rates among staff could not be calculated. We report rate ratios (RR) for individuals incarcerated in Massachusetts relative to the state and US general populations.^5,6^ Analyses were conducted in R, version 3.6.3 (R Foundation).

## Results

At baseline, 14 987 individuals were incarcerated across Massachusetts facilities (MA DOC, 7735; county facilities, 7252). As of July 8, 2020, 1032 confirmed cases of COVID-19 were reported among incarcerated individuals (n = 664) and staff (n = 368). The rate of COVID-19 was 44.3 cases per 1000 persons—2.91 (95% CI, 2.69-3.14) times higher than the Massachusetts general population and 4.80 (95% CI, 4.45-5.18) times the US general population (Table). Reported incidence was lower in county facilities (35.71 cases per 1000 persons) than in MA DOC facilities (52.36 cases per 1000 persons); however, many county facilities had low testing rates (facilities in 5 counties tested <100 per 1000 persons).

**Table.** Rates of Confirmed Cases of COVID-19 and Testing by Carceral System Compared With the General Population in Massachusetts and the United States^a^

Characteristic	United States (n = 329 915 897)	Massachusetts State (n = 6 892 503)	All MA carceral facilities (n = 14 987)	MA DOC: state prison system (n = 7735)	County carceral facilities (n = 7252)
Staff					
Total cases	NA	NA	368	193	175
Proportion of staff among all cases, %^b^	NA	NA	36	32	40
Incarcerated individuals					
Total cases, No.	3 042 503	104 961	664	405	259
Total tested, No.^c^	37 395 666	1 157 023	10 298	8455	1843
Positive tests, %	8	9	6	5	14
Cumulative case rate per 1000 persons	9.22	15.23	44.31	52.36	35.71
Incarcerated population compared with state					
RD (95% CI)	NA	NA	29.08 (25.71-32.45)	37.13 (32.03-42.23)	20.49 (16.14-24.84)
RR (95% CI)	NA	NA	2.91 (2.69-3.14)	3.44 (3.11-3.79)	2.35 (2.07-2.65)
Incarcerated population compared with United States					
RD (95% CI)	NA	NA	35.08 (31.71-38.45)	43.14 (38.04-48.24)	26.49 (22.14-30.84)
RR (95% CI)	NA	NA	4.80 (4.45-5.18)	5.68 (5.14-6.26)	3.87 (3.42-4.37)

Abbreviations: DOC, Department of Corrections; MA, Massachusetts; NA, not applicable; RD, rate difference per 1000 persons; RR, rate ratio.

^a^ All total values are cumulative counts as of July 8, 2020; All rates reported per 1000 persons.

^b^ Cases among staff among all cases (including both staff and incarcerated individuals).

^c^ Excludes antibody tests.

Overall, systems with higher testing rates had higher case rates (Figure, A). For example, the testing rate across all county jails was 254 per 1000 persons, with a case rate of 36 cases per 1000 persons, whereas MA DOC facilities had a testing rate of 1093 per 1000 persons and a case rate of 52 cases per 1000 persons. The proportion of positive tests among incarcerated individuals in county facilities was higher (14%) than in MA DOC facilities (5%) and the general Massachusetts (9%) and US (8%) populations. Case incidence was higher among systems that released a lower proportion of their baseline population (Figure, B). The MA DOC case rate was 52 cases per 1000 persons with a population decrease of 9% compared with all county jails, which had a case rate of 36 case per 1000 persons and decreased their overall population by 21%. County jails released up to 35% of incarcerated individuals. A gradual decline in reported cases among incarcerated individuals and staff was observed, corresponding to a decrease of 15% in the incarcerated population.

**Figure.**
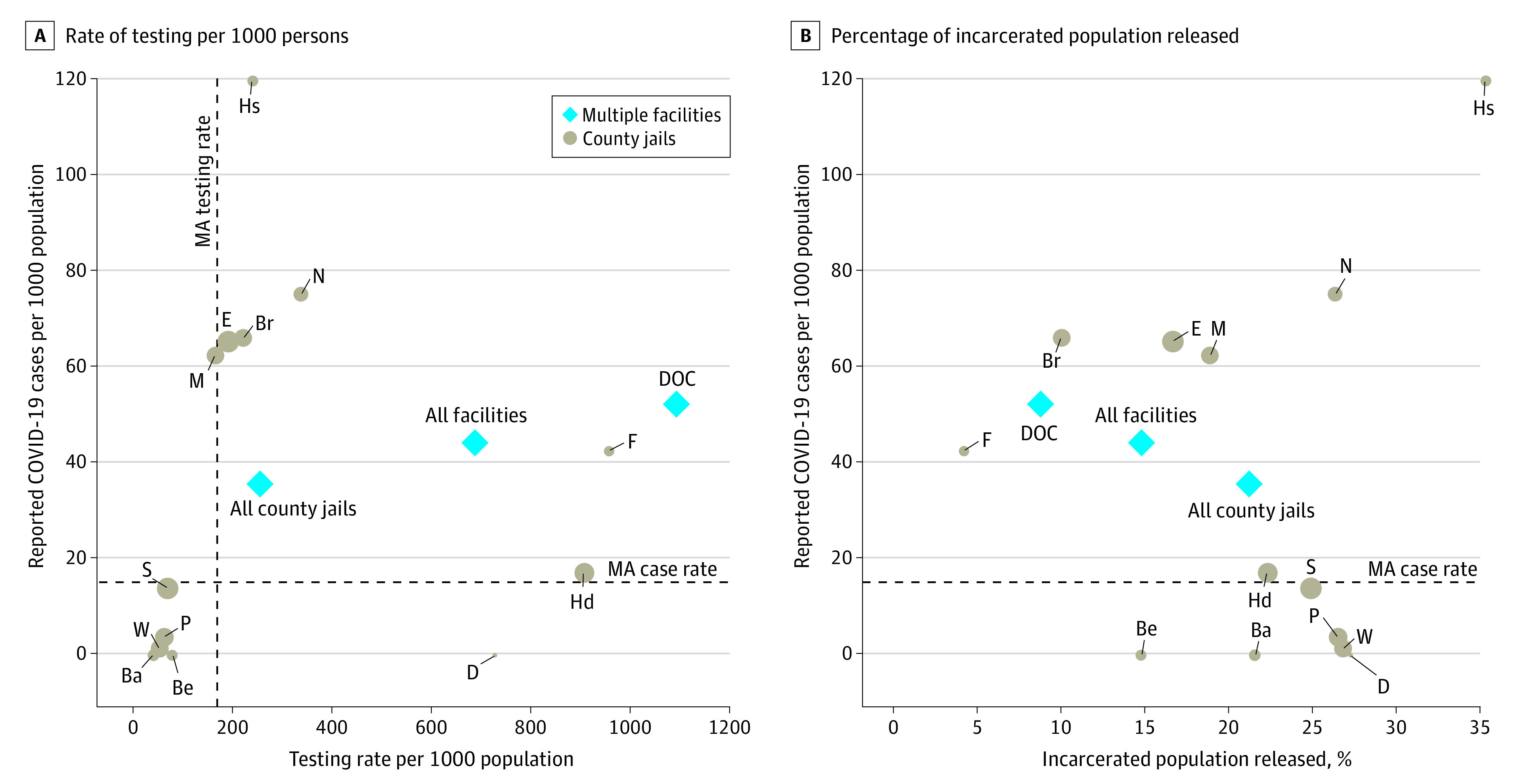
Rates of COVID-19 Cases Compared With the Rates of Testing and the Percentage of the Incarcerated Population Released Rate of (A) COVID-19 testing per 1000 persons and (B) percentage of incarcerated population released against confirmed cases per 1000 among incarcerated persons by county carceral facilities and the state prison system. Diamonds represent multiple facilities; circles, county jails. Ba indicates Barnstable County; Be, Berkshire County; Br, Bristol County; D, Dukes County; DOC, Department of Corrections; E, Essex County; F, Franklin County; Hd, Hampden County; Hs, Hampshire County; M, Middlesex County; N, Norfolk County; P, Plymouth County; S, Suffolk County; and W, Worcester County.

## Discussion

To our knowledge, this is the first examination of COVID-19 burden among incarcerated individuals and staff in both jails and prisons. The rate of COVID-19 among incarcerated individuals was nearly 3 times that of the Massachusetts general population and 5 times the US rate, consistent with recent reports in US federal and state prisons.^2^ Systems with smaller reductions in incarcerated populations and higher testing rates demonstrated higher rates of confirmed cases. Limited testing likely underestimates the true infection rate in county jails, where nearly one-third of tests were positive.

These data are limited by absence of deaths and demographic characteristics. Owing to structural racism and the criminalization of poverty, COVID-19 racial/ethnic inequities may be exacerbated among incarcerated individuals. Whereas rates of COVID-19 vary nationwide, our results add to a growing body of literature emphasizing high COVID-19 rates in carceral settings and the importance of testing and decarceration.^1,2,3^

Rates of COVID-19 in Massachusetts jails and prisons are alarmingly high and require urgent action. Reporting of COVID-19 data from carceral facilities is highly variable and generally excludes county jails; hence, standardization is needed. Access to testing without coercion, decarceration, and contact tracing are necessary to decrease harm from COVID-19 to incarcerated people and their communities.
